# Sensor Fusion in Assistive and Rehabilitation Robotics

**DOI:** 10.3390/s20185235

**Published:** 2020-09-14

**Authors:** Domen Novak, Robert Riener

**Affiliations:** 1 Department of Electrical & Computer Engineering, College of Engineering and Applied Science, University of Wyoming, Laramie, WY 82071, USA; 2 Sensory-Motor Systems Lab, Department of Health Sciences and Technology, ETH Zurich, 8092 Zurich, Switzerland; robert.riener@hest.ethz.ch; 3 Spinal Cord Injury Center, University Hospital Balgrist, University of Zurich, 8008 Zurich, Switzerland

## 1. Introduction

As the world’s population gradually grows older, more and more adults are experiencing sensory–motor disabilities due to stroke, traumatic brain injury, spinal cord injury and other diseases. People with such disabilities can greatly benefit from the help of robotic technologies. For example, rehabilitation robots can help stroke survivors perform the intensive, repetitive exercises needed for relearning motions, robotic prostheses can replace body parts after amputations, and robotic orthoses and exoskeletons can strengthen body parts that have been permanently weakened by disease. Together, all these technologies thus have the potential to greatly improve quality of life for people with disabilities.

However, while the effectiveness of assistive and rehabilitation robots has been demonstrated in several clinical trials (which have found, e.g., that rehabilitation robots are approximately as effective as human therapists), the technology has not yet been broadly adopted by health facilities and end-users. This limited adoption is not due to inappropriate mechanical design—state-of-the-art robots have many degrees of freedom and are mechanically robust to suboptimal operating conditions, allowing them to theoretically deliver support in a variety of real-world environments. Instead, the main limitation is the lack of clarity about how assistive and rehabilitation robots can intelligently recognize and react to both user needs and desires as well as environmental factors in order to provide appropriate support. For example, how can a prosthetic leg recognize the walking terrain and the user’s desired gait speed in order to enable safe, efficient gait? How can an upper body exoskeleton recognize what the user is trying to do (e.g., lift a box) and how can it tailor its assistance to the characteristics of the user (e.g., strength) and task (e.g., box weight)? How can a rehabilitation robot intelligently guide a person with chronic limb impairment through a series of exercises in order to gradually restore limb function over the course of several weeks? Such questions need to be addressed using novel sensor fusion algorithms that can intelligently combine potentially unreliable data from multiple different types of sensors as a basis for decision making and device control.

This Special Issue aims to showcase recent advances in sensor fusion for assistive and rehabilitation robots. It consists of eight papers that present the development and evaluation of exciting technological advances from diverse application areas. It is our hope that the presented technologies will reach clinical evaluation in the next few years and will eventually become widespread in clinical practice and everyday life, improving the quality of life for people with disabilities. Furthermore, we hope that the presented papers will inspire other researchers to conduct further work in this exciting area, thus serving as a foundation for broader research and development.

## 2. Contributions

The first paper, by Huang et al. [1], presents a novel wheelchair robot with attached leg exoskeletons for leg muscle exercise. The authors implemented an exoskeleton control system based on master–slave control and sensor fusion and conducted experiments to evaluate exercise efficiency with regard to the gluteus medius muscles. As many people with lower leg disabilities use wheelchairs, the proposed technology allows them to maintain mobility while also enabling pedaling exercises that can strengthen the limb and prevent atrophy, thus combining assistive and rehabilitation functions.

The second paper, by Kubota et al. [2], presents an evaluation of a lower-limb rehabilitation robot that induced eccentric tibialis anterior muscle contraction by controlling strength and speed using a combination of velocity and force feedback. In a long-term evaluation with 11 elderly participants, the authors found significant differences between training and control phases, though they did not find positive results in a cross-over test. Still, the results demonstrate the feasibility of such biofeedback-based training and indicate directions for the future improvement of lower-limb rehabilitation devices.

The third paper, by Sánchez Manchola et al. [3], presents two gait phase partitioning algorithms based on a single foot-worn inertial measurement unit. Intended for future use with lower-limb exoskeletons, the algorithms are based on either thresholding or hidden Markov models, and are trained and evaluated during treadmill walking tasks. The hidden-Markov-model-based method demonstrated particularly good performance and could potentially become widespread in any technology that requires accurate gait phase partitioning—not just lower-limb exoskeletons, but also full-body exoskeletons and lower-limb prostheses.

The fourth paper, by Farago et al. [4], presents an electromyography-based muscle health model for elbow trauma patients. Surface electromyography recordings were collected from healthy and injured limbs of 30 elbow trauma patients during 10 different motions, and multiple classifiers were used to distinguish between healthy and injured states. This shows the feasibility of using sensor fusion methods to automatically evaluate the health of elbow muscles. In the future, the method could be expanded to allow dynamic monitoring of muscle health as patients progress through the rehabilitation process, and could potentially even be used by intelligent rehabilitation robots to automatically plan therapy exercises.

The fifth paper, by Orand et al. [5], presents a single-subject evaluation of a device for bilateral upper-limb training that incorporates camera-based limb tracking and tactile feedback. The subject underwent six weeks of training, and clinical measures showed pre-post improvement on several scales. Though limited by the small sample size, the results indicate high potential of the presented technology as a low-cost tool for self-administered exercise that could be performed at home or in community settings, resulting in cheaper and more accessible motor rehabilitation as well as a lower burden on clinical rehabilitation facilities.

The sixth paper, by Krausz et al. [6], begins with the hypothesis that the significant gait prediction errors currently seen in robotic lower-limb prostheses are due to the inter- and intra-subject variability of the data sources used for such prediction. They propose the addition of environmental data from a depth sensor worn on the belt, and analyze the variability of such a sensor compared to kinetic, kinematic, and electromyographic data. By demonstrating improvements in separability, repeatability, clustering and desirability across subjects and activities, the authors present a convincing case that the incorporation of vision-based environmental data into lower-limb assistive robots could greatly improve the effectiveness and robustness of such robots.

The seventh paper, by Hlucny and Novak [7] (co-authored by one of the guest editors), presents a proof-of-concept system that uses a combination of inertial measurement units and machine learning to automatically classify different types of human bilateral lifting behaviors. While not yet suitable for real-time use, the proposed method can accurately identify parameters such as the start and end points of a lift and whether the user is lifting in an ergonomic or unergonomic fashion. This information could, in the future, be used to control assistive devices such as trunk exoskeletons for physically demanding occupations, reducing the risk of lower-back injury in, e.g., manual materials handling.

Finally, the eighth paper, by Krausz and Hargrove [8], carries out a survey of teleceptive (remote, contactless) sensing for wearable assistive robotic devices. Related to the aforementioned sixth paper, the authors argue that teleceptive sensing has high potential for providing environmental and contextual awareness that could greatly improve the effectiveness and robustness of assistive robots. They provide a thorough review of different teleception sensor modalities and sensor fusion methods; furthermore, they identify several barriers to clinical translation and suggest several possible research directions, which will help guide further work in this exciting but still fragmented field.
